# Blood biomarker-based classification study for neurodegenerative diseases

**DOI:** 10.1038/s41598-023-43956-4

**Published:** 2023-10-11

**Authors:** Jack Kelly, Rana Moyeed, Camille Carroll, Shouqing Luo, Xinzhong Li

**Affiliations:** 1 Faculty of Medicine, Biology and Health, Centre for Biostatistics, School of Health Sciences, University of Manchester, Manchester, UK; 2 Faculty of Health, University of Plymouth, Plymouth, PL6 8BU UK; 3 Faculty of Science and Engineering, University of Plymouth, Plymouth, PL6 8BU UK; 4 School of Health and Life Sciences, Teesside University, Middlesbrough, TS1 3BX UK

**Keywords:** Dementia, Parkinson's disease, Predictive markers

## Abstract

As the population ages, neurodegenerative diseases are becoming more prevalent, making it crucial to comprehend the underlying disease mechanisms and identify biomarkers to allow for early diagnosis and effective screening for clinical trials. Thanks to advancements in gene expression profiling, it is now possible to search for disease biomarkers on an unprecedented scale.Here we applied a selection of five machine learning (ML) approaches to identify blood-based biomarkers for Alzheimer's (AD) and Parkinson's disease (PD) with the application of multiple feature selection methods. Based on ROC AUC performance, one optimal random forest (RF) model was discovered for AD with 159 gene markers (ROC-AUC = 0.886), while one optimal RF model was discovered for PD (ROC-AUC = 0.743). Additionally, in comparison to traditional ML approaches, deep learning approaches were applied to evaluate their potential applications in future works. We demonstrated that convolutional neural networks perform consistently well across both the Alzheimer's (ROC AUC = 0.810) and Parkinson's (ROC AUC = 0.715) datasets, suggesting its potential in gene expression biomarker detection with increased tuning of their architecture.

## Introduction

Blood tissue is a convenient and accessible source and alterations in blood gene expression study reflecting the progression of disease progression^1^. This makes transcriptomics data an important type of omics data type for the diagnostic investigation of human diseases. However, there is a lack of reliable blood-based biomarkers for the non-invasive diagnosis either of both Alzheimer's disease (AD) or Parkinson's disease (PD). α-synuclein and DJ-1 proteins have been investigated as blood biomarkers for PD and demonstrated a high potential to be used in the clinic^2–4^, however, the validation of both failed in further studies^5–8^. No blood biomarkers for AD have been used clinically to date, with research identifying Aβ42/40 ratio levels^9^ and Neurofilament Light Chain (NfL) blood concentration as future potential^10,11^.

Blood gene biomarkers for neurodegenerative diseases (NDs) are particularly interesting as they have high accessibility and are relatively cheap to perform. However, identifying ND gene expression biomarkers in blood with good reproducibility has been difficult in the past due to the small sample sizes of available datasets^12^. The use of statistical learning has been of particular interest for investigating blood gene biomarkers due to the high dimensionality of gene expression data. Machine learning (ML) algorithms can be used for feature selection to identify gene panels that can be subsequently used to distinguish between disease and control patients. This panel of genes can then be used to train classification models that can identify or predict disease status. Long et al.^13^ applied a support vector machine (SVM) to a small AD dataset and but returned good results. Later on, they worked on a larger dataset using the least absolute shrinkage and selection operator (LASSO) feature selection approach and SVM classifier, discovered a good classification model with a receiver operating characteristic (ROC) area under the curve (AUC) of 0.87. More recently, Lee and Lee^14^ used multiple feature selection together with classification algorithms on multiple datasets and identified models that worked well within different datasets but performed poorly between datasets. Shamir et al.^15^ conducted the largest gene expression analysis of PD tissue in the whole blood, including 205 PD patients and 233 health patients. They used an SVM approach to classify PD patients from healthy controls using 87 gene signatures and achieved a ROC AUC performance of 0.79. Wang et al.^16^ reanalysed the same dataset, taking a random forest (RF) approach to classify PD patients from healthy control, and achieved a lower ROC AUC of 0.74. With the limited classification approaches used on such a relatively large dataset, there is great potential for investigating other methodologies to improve the classification performance.

In this study, we collected the largest publicly available blood transcriptomics datasets and built robust and replicable models for AD and PD studies respectively by applying a range of feature selection and classification approaches.

## Methodology

### Data processing

**Figure 1:**
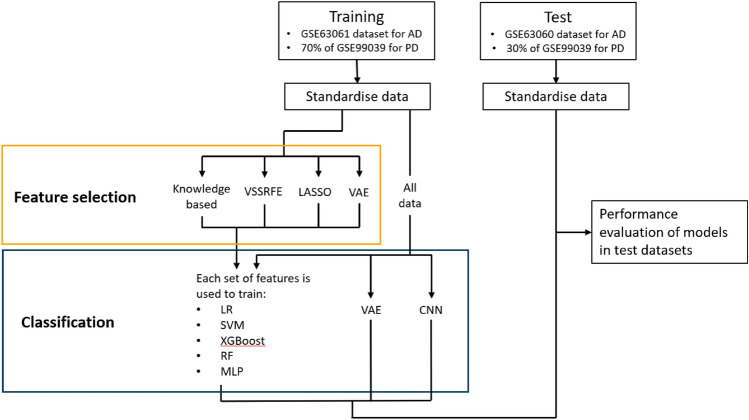
Workflow for identification of blood biomarkers. Training and test datasets were standardized separately. Feature selection is applied to training data to generate feature sets of genes using different approaches. Each of these feature sets is used to train five different classification models to distinguish control and disease patients: linear regression (LR), support vector machine (SVM), XGBoost, random forest (RF), and multilayer perceptron (MLP). The feature set and classification model combinations are evaluated in test datasets. Additionally, VAE and convolutional neural network (CNN) models are trained and evaluated separately.

The full workflow of this study is shown in Fig. 1. The publicly available AD peripheral venous whole blood datasets were downloaded from the Gene Expression Omnibus database (GEO http://www.ncbi.nlm.nih.gov/geo/ ) with accession identifiers GSE63061 and GSE63060. As these two independent datasets were generated from the AddNeuroMed cohort study^25^ with the same sample collection and analysis protocols, they were used as independent training and test datasets respectively in our study. They were processed separately using the same methodology described previously^26^, however, the mild cognitive impairment (MCI) patients were removed from each dataset to reduce study complexity. Additionally, since low-expression genes have been shown to be important features in previous machine learning-based microarray analyses^27^, the bottom 5% of probes by average expression value in datasets were not discarded. Meanwhile, the publicly available peripheral.

Venous whole blood dataset comprising 205 PD and 233 healthy control samples were downloaded from the GEO with accession identifier GSE99039. This dataset was processed using the previous methodology^26^ without removing the bottom 5% of probes by average expression value. This dataset was randomly divided into a training and a testing dataset so that 70% of samples were for training and 30% for testing. This process was done by using the train_test_split() function in the python sklearn library^28^.

All AD and PD datasets were scaled using StandardScaler() in sklearn, which transforms each feature distribution to a mean value of 0 and a standard deviation of 1. This was done in training and testing datasets separately. t-distributed stochastic neighbor embedding (t-SNE) was used to visualise local structures of the high-dimensionality data and identify any clear groups by dimensionality reduction. t-SNE was created using the TSNE() package in sklearn and 5 runs with perplexity set to 5, 15, 30, 40 and 50 run over 1000 iterations. They were then visualized using the tsneplot() function in bioinfokit (v0.9)^29^.

### Feature selection

The following multiple feature selection approaches were considered in the model training process:

- Knowledge-based feature selection
- Variable step size recursive feature elimination (VSSRFE) with Logistic regression
- LASSO
- Variational autoencoder (VAE)

Five-fold cross-validation (CV) was performed to optimise the precision-recall AUC (prAUC) in the feature selection process that was done using BayesSearchCV() in the scikit-optimize python library. prAUC was used for optimisation as it is less sensitive to unbalanced classes^30^. The selected subset of features was used later in the training and test datasets to apply different ML algorithms.

#### Knowledge-based feature selection

To investigate whether feature selection within the context of existing biological knowledge can improve classification performance and yield better-classifying models, a set of genes based on previous knowledge of the disease was included. In addition, genes with high variances across all samples are included as well.

For the PD dataset, the following sources were used to identify knowledge-based genes:

- DEGs identified in a previous meta-analysis^1^ (1046 genes).
- Genes in control network modules not preserved in PD networks described in our previous paper^26^ (606 genes).
- Genes in PD network modules not preserved in control networks described in our previous paper^26^ (651 genes).
- Genetic risk genes from Genome-Wide Association Studies (GWAS)^31^ (70 genes).
- Genes from the KEGG pathway of 'KEGG PARKINSONS DISEASE'^32,33^ (128 genes).

In addition to these, the top 3000 genes by median absolute deviation (MAD) in the PD training dataset were included as well.

For the AD dataset, the following sources were used to identify knowledge-based genes:

- DEGs identified in our previous meta-analysis of AD frontal cortex^34^ (3124 genes).
- Genes in control network modules not preserved in AD networks described in our previous paper^26^ (1019 genes).
- Genes in AD network modules not preserved in control networks described in our previous paper^26^ (1076 genes).
- AD GWAS genes^35^ (30 genes).
- Genes from the KEGG pathway of 'KEGG ALZHEIMERS DISEASE' pathway^32,33^ (165 genes).
- Risk genes from the Alzgene database^36^ (680 genes).

In addition to these, the top 3000 genes by MAD in the AD training dataset were included.

#### Recursive feature elimination with variable step size

Variable step size recursive feature elimination (VSSRFE) works to recursively eliminate the most unimportant feature until a feature set remains, as described by Li et al*.*^37^. Briefly, an estimator is trained to find the importance of features in the dataset and the least important features are removed. The number of features removed at the first step is determined by the initial step size, and as the number of features in the dataset is halved, the step size is also halved until the step size is one. This is repeated recursively on the feature set until the data is pruned to the desired number of features, usually the number that gives the best performance evaluation scores from the estimator. As the samples included in all datasets are relatively similar, the initial step size is set to 100 for all. The feature weights used in VSSRFE are confirmed by using linear regression (LR). The parameter controlling the strength of regularization of LR is tuned on the whole training datasets before VSSRFE using Bayesian optimization with a fivefold CV.

#### Feature reduction using LASSO

LASSO and elastic net reduce the number of features using regularisation. Regularisation approaches to feature selection can shrink some coefficients of features to zero and remove these features from the model. The LASSO algorithm was applied with the sklearn python library to reduce the dimensions of the data. The α constant that multiplies the L1 term was optimized so that the full feature set was reduced to the best subset of features.

#### Variational autoencoder (VAE)

As microarray data generally has a high dimensionality with a large number of features and relatively low sample numbers, VAE has great potential to reduce the dimensionality of data^38^. The basic VAE architecture applied here was based on the VAE proposed by Zhang et al.^38^. The encoder reduces the number of features to 128 at the latent space, which was used with the ML classification algorithms.

The VAE is built using the Keras module in python^39^ with each layer using a Rectified Linear Unit (ReLU) activation function and compiled using an Adam optimiser and categorical cross-entropy loss function with the early stopping of 10, so if the loss function does not improve across for 10 epochs the training is stopped. The optimum VAE architecture was confirmed using five-fold cross-validation (5-fold CV), identifying the model with the best average accuracy from its softmax classifier.

Three architectures of VAE were tested:

- Basic VAE architecture based on the VAE from Zhang et al.^38^
- Basic VAE architecture including batch normalization at each layer of the VAE.
- Basic VAE architecture including batch normalization at each layer of the VAE and dropout layers of 20% to prevent overfitting.

### Machine learning for classification

Optimization of classification algorithms was performed on training datasets using Bayesian optimization with fivefold CV to optimize the prAUC. Supplementary table S3 shows the classification algorithms used on various feature sets for PD and AD training datasets. It also shows the base python code to run the algorithms and the parameters that are tuned to optimize the algorithm to training data. These algorithms were tuned and trained on all features in the training datasets and the feature sets found using the four feature selection methods discussed above to identify which feature set each classification method performs best on. In addition to these approaches, neural network approaches that have built-in dimensionality reduction and classification were used. The VAE architecture was used to reduce the feature down to 128 and softmax classifier to assign samples as diseases or controls. A convolutional neural network (CNN) model was built as well based on CNN applications in computer vision^23^. CNNs are similar to multilayer perceptrons (MLPs), however, have some changes that make them effective when using multiple layers and good at reducing data dimensionality^22^. To start, gene expression data is reshaped to a two-dimensional space to be like image data. After a two-dimensional convolutional layer, a ReLU activation function is applied. This data is then passed to a maxpooling layer and flattened before it is passed to a dense layer with a ReLU activation function. Softmax is then used as a classifier. This CNN is compiled using a stochastic gradient descent (SGD) optimizer and categorical cross-entropy loss function in the Python Keras package.

Supplementary Table S1a and S1b summarize the parameters used in ML approaches and datasets information respectively. The performance of all classification models was assessed using ROC-AUC (plotted using the roc curve function in the sklearn python package) and prAUC (plotted using precision recall curve function in sklearn). All relevant python code used for this study is available at https://doi.org/10.5281/zenodo.4483751.

## Results

### Data processing

After pre-processing the GSE99039 PD dataset was randomly split into a training dataset of 141 PD and 162 controls and a test dataset of 68 PD and 63 controls, all of which initially had 20,183 features. The GSE63061 dataset being used as the training dataset for the AD study included 137 AD and 131 control samples, and the test dataset (GSE63060) included 143 AD and 104 controls, all initially had 19,147 features. Local structures in the datasets and outlier samples were identified by reducing dimensionality using t-SNE. The t-SNE plots with a perplexity of 30 are shown in Supplementary Figure S1 indicating no outliers in the data. The perplexity of 30 was chosen as it gave the clearest visualization of the data.

### Feature selection

Four approaches to feature selection were used on the AD and PD training datasets to identify the best biomarker panel of genes used in classification models. The number of features identified in each approach was shown in Table 1.

**Table 1:** The number of features identified using each feature selection approach.

Feature selection approach	Number of features identified in each dataset	
	PD	AD
Knowledge based feature selection	4981	7520
VSSRFE	5	159
LASSO regularisation	19	2
VAE	128	128

#### Knowledge-based feature selection

To investigate whether they yield better classification models, a set of features based on existing biological knowledge was used. In the PD dataset, a combination of the 2500 knowledge-based features and 3000 highest MAD features returned 4981 unique features mapped in the PD dataset. In the AD dataset, a combination of the 5953 knowledge-based features and 3000 highest MAD features returned 7520 unique features mapped in the AD dataset.

#### Recursive feature elimination with variable step size

**Figure 2:**
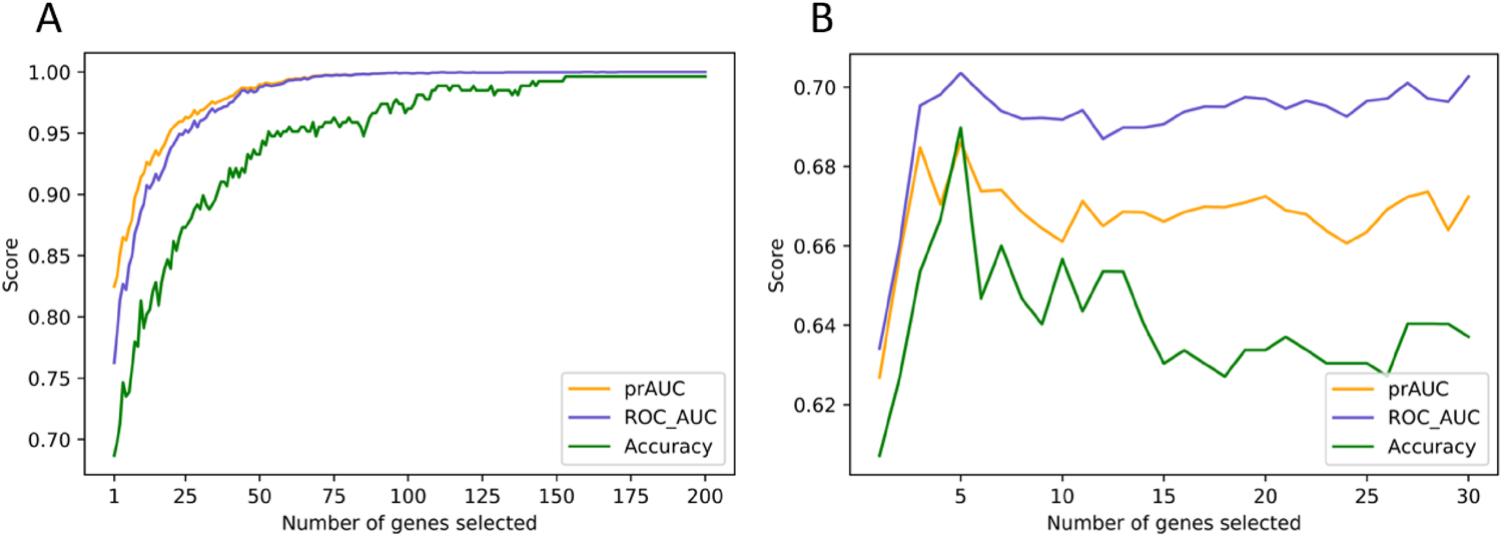
Evaluation scores for different numbers of genes selected using VSSRFE. VSSRFE identified a panel of 159 genes on AD data (**A**) and a panel of 5 genes on PD data (**B**) that gave the best prAUC, ROC-AUC, and accuracy scores.

Feature weights used in VSSRFE were discovered by using LR. On AD training data VSSRFE identified a panel of 159 genes which gave a prAUC, ROC-AUC, and accuracy of 1.00 (see Fig. 2A). On PD training data, VSSRFE identified a panel of 5 genes (*DGKK, PTGDS, LSP1, PDLIM7,* and *KIR2DL3*) that gave the maximum prAUC of 0.686, ROC-AUC of 0.704, and accuracy of 0.690 (see Fig. 2B).

#### Feature reduction using LASSO

The number of features was reduced by regularization using LASSO. LASSO identified a gene set of 2 genes (*NDUFS5, RPL36AL*) that gave the best model (prAUC = 0.8191) for the AD dataset. Using the PD dataset, LASSO identified a gene set of 19 genes that gave the best but a poor model (prAUC = 0.5861).

#### Variational autoencoder (VAE)

The number of features was reduced to 128 using a VAE model. On the AD training data, the basic VAE architecture performed the best (accuracy of 0.623) over VAE with batch normalization (accuracy of 0.537) and dropout layers (accuracy of 0.560). The learning rate for the VAE was reduced to 0.00001 when the model did not converge at 0.001. On the PD training data, the VAE with batch normalization and dropout layers gave the best accuracy (0.554), though not much better than either VAEs without dropout, which both had an accuracy of 0.548.

### Machine learning for classification

Optimization of all classification algorithms was performed using the Bayesian optimization approach with fivefold CV. Five classification algorithms (LR, SVM with radial kernel, RF, XGBoost^17^, MLP) were optimized and run using each of the gene sets identified by above the feature selection approaches. Additionally, a VAE and a CNN were optimized and used to reduce dimensionality and classify data.

**Figure 3:**
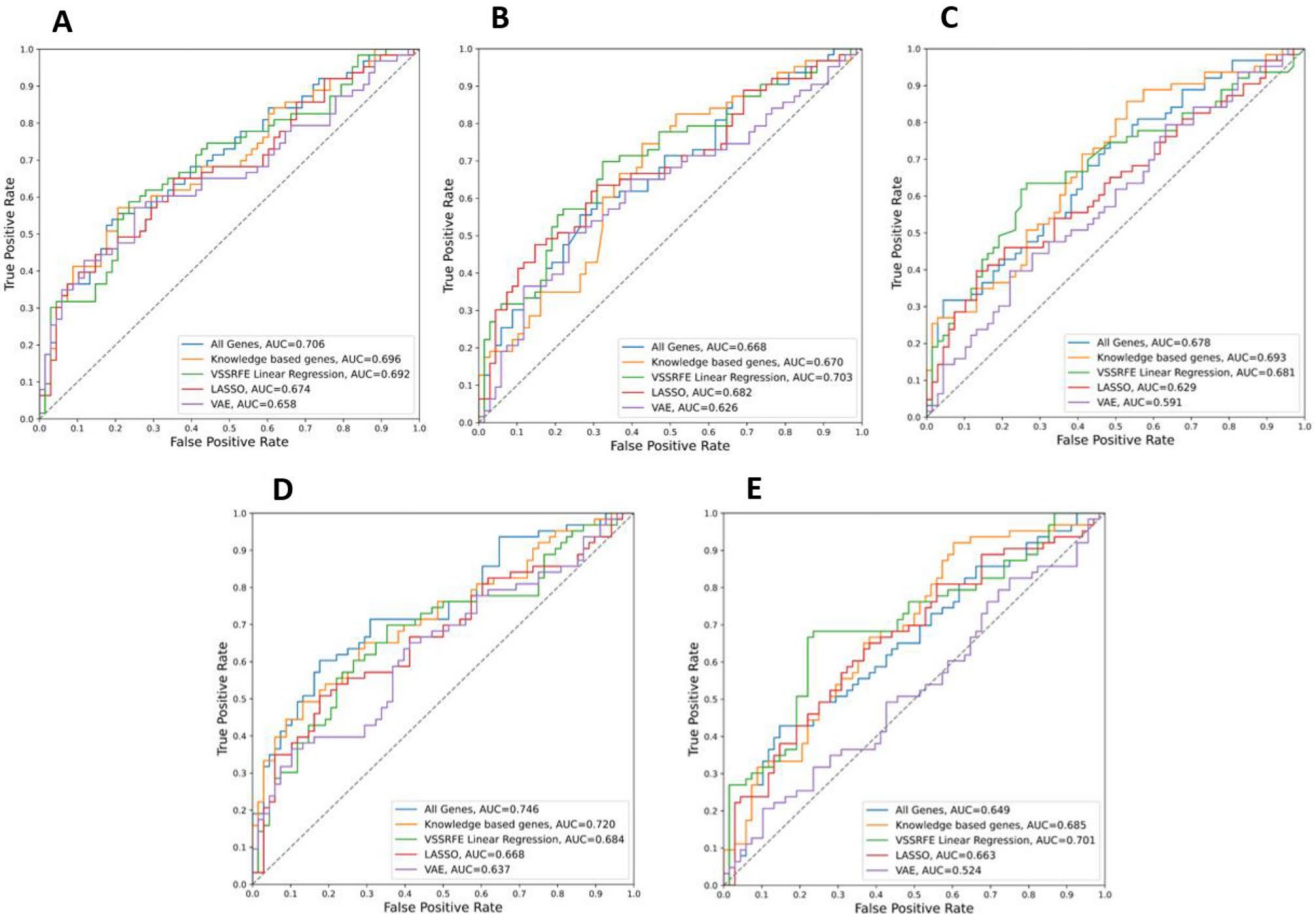
ROC curves for each classification algorithm on PD data.

For the PD dataset, the evaluation scores of each classification algorithm were shown in Table 2. The results for each classification approach using all feature sets identified in feature selection is shown. There were 20,183 genes in the PD dataset and 4981 in the knowledge genes feature set. VSSRFE feature selection selected 5 features (*DGKK*, *PTGDS*, *LSP1*, *PDLIM7* and *KIR2DL3*), LASSO selected 19 features and VAE reduced all features to a representative 128 features. CNN and VAE classifiers inherently reduce feature dimensions so do not require feature selection. The ROC curves for each classification algorithm were shown in Fig. 3. All models except one (MLP using VAE feature selection) had an accuracy higher than the proportion of the largest observed class (non-information rate) of the test data (0.519). The RF model trained using all genes gave the best accuracy (0.702), ROC AUC (0.743), and prAUC (0.762), however, had a much lower sensitivity (0.571) than specificity (0.824).

**Table 2:** Evaluation of classification algorithms on PD data.

Classification algorithm	Feature selection	Accuracy	Sensitivity	Specificity	ROC-AUC	prAUC
LR	All genes	0.664	0.571	0.750	0.706	0.714
	Knowledge genes	0.672	0.540	0.794	0.696	0.710
	VSSRFE	0.672	0.587	0.750	0.692	0.681
	LASSO	0.641	0.571	0.706	0.674	0.682
	VAE	0.656	0.587	0.721	0.658	0.694
SVM	All genes	0.626	0.476	0.765	0.668	0.668
	Knowledge genes	0.565	0.397	0.721	0.670	0.665
	VSSRFE	0.641	0.460	0.809	0.703	0.696
	LASSO	0.641	0.556	0.721	0.682	0.689
	VAE	0.618	0.540	0.691	0.625	0.592
XGBoost	All genes	0.588	0.556	0.618	0.678	0.679
	Knowledge genes	0.618	0.524	0.706	0.693	0.698
	VSSRFE	0.679	0.603	0.750	0.681	0.675
	LASSO	0.588	0.556	0.618	0.629	0.642
	VAE	0.550	0.540	0.559	0.591	0.567
RF	All genes	0.702	0.571	0.824	0.743	0.762
	Knowledge genes	0.672	0.476	0.853	0.716	0.737
	VSSRFE	0.672	0.603	0.735	0.684	0.682
	LASSO	0.641	0.556	0.721	0.668	0.671
	VAE	0.573	0.508	0.632	0.637	0.666
MLP	All genes	0.603	0.540	0.662	0.649	0.609
	Knowledge genes	0.618	0.540	0.691	0.685	0.663
	VSSRFE	0.672	0.556	0.779	0.701	0.684
	LASSO	0.626	0.556	0.691	0.663	0.626
	VAE	0.511	0.444	0.574	0.517	0.504
CNN		0.695	0.667	0.721	0.715	0.710
VAE		0.672	0.556	0.779	0.713	0.712

**Figure 4:**
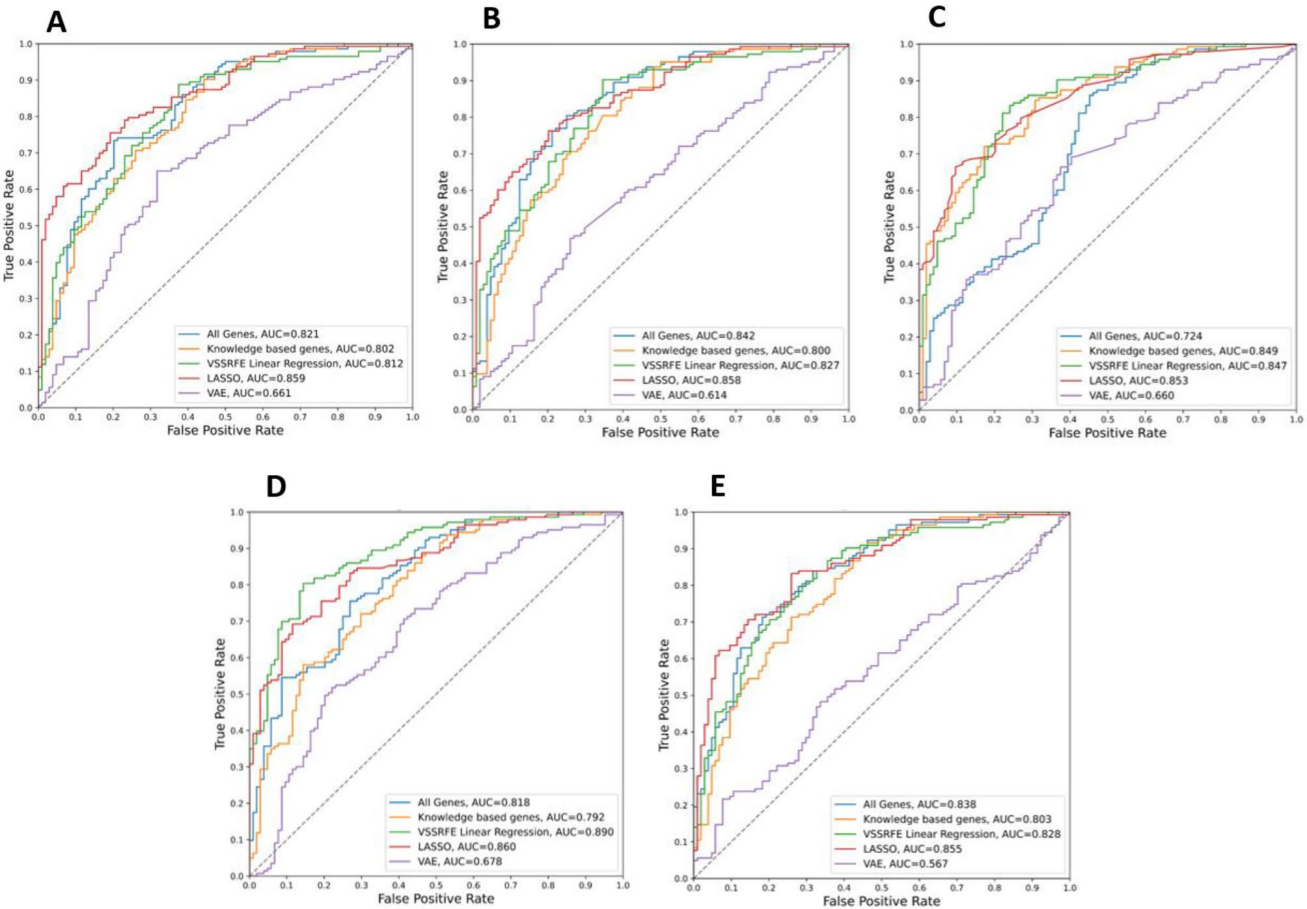
ROC curves for each classification algorithm on AD data.

This may be advantageous for biomarkers as a false negative diagnosis is much preferred to a false positive. The CNN model performed well with consistently high scores across all evaluation approaches. The evaluation scores of each classification algorithm for the AD dataset were shown in Table 3. The ROC curves for each classification algorithm were shown in Fig. 4. All models except two (MLP and SVM using VAE feature selection) had an accuracy higher than the non-information rate of the test data (0.579). The RF model trained using the 159-feature panel identified by VSSRFE gave the best accuracy (0.810), ROC AUC (0.889), and prAUC (0.919). Supplementary Tables 1 & 2 demonstrated the confusion matrix summarising the performance of AD and PD studies respectively.

**Table 3:** Evaluation of classification algorithms on AD data.

Classification algorithm	Feature selection	Accuracy	Sensitivity	Specificity	ROC-AUC	prAUC
LR	All genes	0.737	0.811	0.635	0.821	0.848
	Knowledge genes	0.713	0.783	0.615	0.802	0.830
	VSSRFE	0.733	0.755	0.702	0.812	0.842
	LASSO	0.777	0.790	0.760	0.859	0.899
	VAE	0.648	0.657	0.635	0.661	0.692
SVM	All genes	0.769	0.853	0.654	0.842	0.860
	Knowledge genes	0.737	0.797	0.654	0.800	0.822
	VSSRFE	0.745	0.769	0.712	0.827	0.858
	LASSO	0.769	0.797	0.731	0.858	0.898
	VAE	0.579	0.497	0.692	0.615	0.661
XGBoost	All genes	0.599	0.599	0.654	0.724	0.764
	Knowledge genes	0.741	0.713	0.779	0.841	0.875
	VSSRFE	0.794	0.853	0.712	0.847	0.883
	LASSO	0.725	0.587	0.913	0.858	0.902
	VAE	0.628	0.839	0.337	0.660	0.709
RF	All genes	0.741	0.748	0.731	0.820	0.855
	Knowledge genes	0.700	0.720	0.673	0.792	0.820
	VSSRFE	0.810	0.818	0.798	0.889	0.919
	LASSO	0.717	0.573	0.913	0.860	0.903
	VAE	0.656	0.790	0.471	0.678	0.684
MLP	All genes	0.761	0.839	0.654	0.838	0.873
	Knowledge genes	0.721	0.790	0.625	0.803	0.829
	VSSRFE	0.757	0.804	0.692	0.828	0.863
	LASSO	0.765	0.720	0.827	0.855	0.890
	VAE	0.514	0.378	0.702	0.567	0.659
CNN		0.765	0.895	0.587	0.810	0.845
VAE		0.757	0.923	0.529	0.798	0.816

The confusion matrices of the best models for PD and AD datasets are shown in Supplementary Table S2 and S3 respectively. The confusion matrices for each model are shown in Supplementary Figure S2 and S3 for PD and AD datasets respectively.

### Ethical approval

No ethical approval was needed.

## Discussion

The diagnosis of AD and PD are still challenging tasks in clinical practice, partly due to the lack of accessible and accurate blood biomarkers. Here, different classification algorithms were applied to transcriptomics data to identify a panel of genes and an optimal ML model that has the potential as a prediction model for ND.

Using a diverse variety of feature selection and ML approaches the best-performing models were identified for AD and PD respectively. The best-performing model for PD was RF with all genes included (accuracy = 0.702, ROC AUC = 0.743, prAUC = 0.762). The best AD model using a RF model with a 159 gene panel performed better than PD model (accuracy = 0.810, ROC AUC = 0.889, prAUC = 0.919). Many previous AD studies using ML to identify biomarkers have utilised small datasets^12^. A recent study by Lee and Lee^14^ tested various feature selection and classification approaches to three AD datasets. The highest ROC AUC of 0.874 was identified by using a deep neural network with DEGs with a high convergent functional genomics score^18^.

Many of the models built in this work outperformed previous studies. The RF model trained on the feature set identified by VSSRFE with the LR algorithm gave very promising results with the best accuracy (0.810), ROC AUC (0.889), and prAUC (0.919). This model had a relatively balanced sensitivity and specificity (0.818 vs. 0.798). High specificity was found in other models as well with several models having a specificity of 0.913, however, this came at the cost of lower sensitivity. The set of 159 features has the potential as a diagnosis panel for AD but needs further validation.

Various previous studies have identified blood-based gene expression variations and signatures associated with PD. Jiang et al.^19^ performed feature selection on PD blood transcriptomics data by identifying DEGs, reducing dimensions using LASSO, and then performing recursive feature addition with a SVM on the remaining features. They identified a panel of 9 genes (*PTGDS, GPX3, SLC25A20, CACNA1D, LRRN3, POLR1D, ARHGAP26, TNFSF14,* and *VPS11*) which were used with SVM, RF, and decision tree model classifiers. *PTGDS* and *LRRN3* were the only genes from their feature set that was in any of the feature selection method used in this study, with the former being identified in all feature selection approaches and the latter only present in genes based on previous knowledge. They identified the best classification approach to be RF with a ROC AUC of 0.777, however, this study has many limitations. Their limited approach to feature selection that involved only using DEGs likely removed many key features early before LASSO could be applied. The largest limitation of their study is the small size of the test dataset, which can introduce bias that results in performance estimations that do not reflect the true quality of the model. Work by Shamir et al.^15^, who achieved a ROC AUC greater than those found in this study using the same dataset, also had this limitation. Falchetti et al.^20^ used much larger test datasets by performing a meta-analysis of four PD blood datasets. For feature selection, they selected the top 100 DEGs by absolute effect size and used RFE to identify a gene set of 59 genes that was used with 9 classification algorithms. Using an 80% training and 20% test split of the data they had a more balanced split of data than previous studies. The best model they identified was an SVM with a radial kernel which achieved a ROC AUC of 0.791, although many of their models outperformed those created in this study. Datasets used by Falchetti et al. were combined by merging after re-scaling each gene in each dataset which, although made the sample size much greater, may have introduced covariates to the data, especially with high levels of technical noise present in microarrays.

In our study, the large sample size of the train and test data that have come from the same study avoided many of the limitations that these previous studies have had. Although our PD models underperform compared to previous results, the larger sample size increases the likelihood that the results are reproducible, which is extremely important for diagnostic study. In addition, our PD model had low sensitivity but high specificity which means that patients who do not have the disease are not misdiagnosed or over-diagnosed.

Our study revealed that the VAE feature selection approach performed relatively poorly at capturing a representation of gene expression pattern that can be used for classification. Other previous research has also shown that VAE approaches lose important information in ND study^14^. This is likely due to the complex nature of gene expression patterns in blood for NDs. However, in solid tissue studies where the impacted tissue can be directly biopsied such as skin cancer, VAE has been demonstrated as an effective way of reducing feature dimensionality while retaining feature information^21^, it is not practical in NDs studies. Traditionally applied to imaging data, CNNs work well with many layers making them suitable for reducing data dimensionality and classification^22^. Previously, they have been shown to work well in classifying various cancer types^23^. In our blood-based gene expression AD study, the CNN had high sensitivity which makes them good for detecting actual cases of the disease, however, they also have a high rate of false positives. In the case of early detection, high sensitivity is important so patients with ND are not missed, and any false positives can often be ruled out by further testing by healthcare professionals.

The results from our study describe the potential diagnostic application for NDs, however, there are some limitations. Studies to identify gene expression biomarkers require very large sample sizes to identify a reliable signature for diagnosis^15^. The datasets used in this study are the largest that are publicly available and so should give comprehensive results, however, would likely require validation in thousands of samples^15^. Information on patients' disease history and symptoms would make it possible to investigate the effect of ND as the disease progresses and develop prognostic biomarkers. This could make it possible to create biomarkers that predict the risk of developing certain symptoms. Moreover, it would be beneficial to have phenotypic data on individuals including their age, gender, smoking status, and other variables that could influence the development of diseases. Furthermore, information that could impact data collection and processing, such as the relative abundance of blood cell types in samples, would also be valuable.

Misdiagnosis rates in ND are very high, for instance, misdiagnosis rates of AD range from 12 to 23% in pathologically confirmed studies^24^. Diagnosis of ND is generally based on clinical examination and ruling out other potential causes of symptoms using brain imaging and blood tests. As a result of this, there is also the potential that patients in the cohorts with which the ML models are being trained and tested are actually misdiagnosed. If misdiagnosed patients are included in the initial study, it is likely the models will continue to misdiagnose patients with similar conditions. In the datasets used in this study, diagnosis criteria are stricter than the minimum requirement for diagnosis in clinical settings, which should reduce the impact of this on the results of this work.

This study aimed to build blood-based gene expression prediction models for AD and PD. Additionally, it aimed to assess whether feature selection under the context of existing biological knowledge can contribute to improving classification performance. The feature selection and classification approaches used in this study are the most thorough ND dataset to date. Our classification models have successfully classified AD and PD patients from controls with very good evaluation metrics and show potential promise for ND in clinical practice. The potential of deep learning, particularly CNNs, was also investigated which can be improved and refined in further studies. There is still potential for more data-driven approaches including feature weighting that would likely improve feature selection and classification. Additionally, ensembled models could potentially enhance predictive capabilities by combining the strengths and leverage the diversity of multiple algorithms. Future work, particularly on larger datasets, an ensembled model approach could be taken.

## Conclusion

In this study, we pursued blood-based biomarkers for AD and PD using machine learning and gene expression profiling. Our research yielded promising results, with the best AD model achieving an accuracy of 81%, a ROC AUC of 0.889, and a precision-recall AUC of 0.919 while the best PD model reached a ROC AUC of 0.743. Notably, deep learning algorithms, specifically CNNs, exhibited consistent performance across both AD and PD datasets, highlighting their potential in gene expression biomarker detection. This study underscores the potential for non-invasive, blood-based biomarkers to revolutionize early diagnosis and management of AD and PD, though further research with larger datasets and patient history integration is needed for robust validation and deeper insights into disease progression.

### Supplementary Information

Supplementary Information.

## Data Availability

The datasets applied in this study are cited in the main text, e.g., they are either from the GEO website or from the cited references. The code used to generate results is open access at https://doi.org/10.5281/zenodo.4483751.
