# Supplementary material for: Blood biomarker-based classification study for neurodegenerative diseases
Source: Sci Rep. 2023 Oct 11;13:17191. doi: 10.1038/s41598-023-43956-4 (PMC10567903; doi:10.1038/s41598-023-43956-4)
Supplement: Supplementary file 1 — Supplementary Information. [file 41598_2023_43956_MOESM1_ESM.pdf]

Supplementary Materials

Blood biomarker-based classification study for neurodegenerative diseases

Table S1a: Classification models and the parameters that were tuned on training data

Classification algorithm	Python library	Base python code	parameters tuned
LR	sklearn (28)	LogisticRegression(random state=2, class weight=’balanced’, penalty=’l2’, solver=’liblinear’)	• C
SVM with radial kernel	sklearn (28)	SVC(random state=142, kernel = ’rbf’, class weight = ’balanced’)	• C<br>• Gamma
XGBoost	xgboost (17)	XGBClassifier(random state=42)	• scale_pos_weight<br>• learning_rate<br>• n_estimators<br>• max_depth<br>• min_child_weight<br>• gamma<br>• colsample_bytree<br>• subsample<br>• reg_alpha<br>• reg_lambda
RF	sklearn (28)	RandomForestClassifier(random state=10, class weight=’balanced’)	• max_depth<br>• min_samples_leaf<br>• n_estimators<br>• min_samples_split<br>• max_features
MLP	sklearn (28)	MLPClassifier(random state=10, max iter = 10000, tol = 0.00001)	• activation<br>• hidden_layer_size<br>• solver
VAE	Keras (39)	model.compile(optimizer=’adam’, loss=’categorical crossentropy’, metrics=[metrics.AUC(name=’PR’, curve =’PR’)])	• batch normalisation<br>• dropout layers
CNN	Keras (39)	model.compile(loss = ‘categorical crossentropy’, optimizer = ‘sgd’, metrics = [‘categorical accuracy’])	• dense layer size<br>• filters<br>• kernel size

Table S1b: Information about each study included in this study. The GSE99039 dataset did not have complete sex and age data.

GEO Accession number	Condition		Male	Female	All	Age range (Average)
GSE63061	AD (train)	AD	54	83	137	59-95 (78.0)
		Control	52	79	131	68-91 (75.4)
GSE63060	AD (test)	AD	45	98	143	58-88 (75.4)
		Control	42	62	104	52-87 (72.4)
GSE99039	PD	PD	N/A	N/A	204	N/A
		Control	N/A	N/A	230	N/A

Table S2: Confusion matrix summarising the performance of a best classification model on PD data. The RF model trained using all 20183 genes in the dataset gave the best evaluation scores (accuracy = 0.702, ROC AUC = 0.743, prAUC = 0.762)

	True Positive	True Negative	Total
**Predicted Positive**	56	12	68
**Predicted Negative**	27	36	63
**Total**	83	48	131

Table S3: Confusion matrix summarising the performance of a best classification model on AD data. The RF model trained using the 159 features identified using VSSRFE gave the best evaluation scores (accuracy = 0.810, ROC AUC = 0.889, prAUC = 0.919)

	True Positive	True Negative	Total
**Predicted Positive**	83	21	104
**Predicted Negative**	26	117	143
**Total**	109	138	247

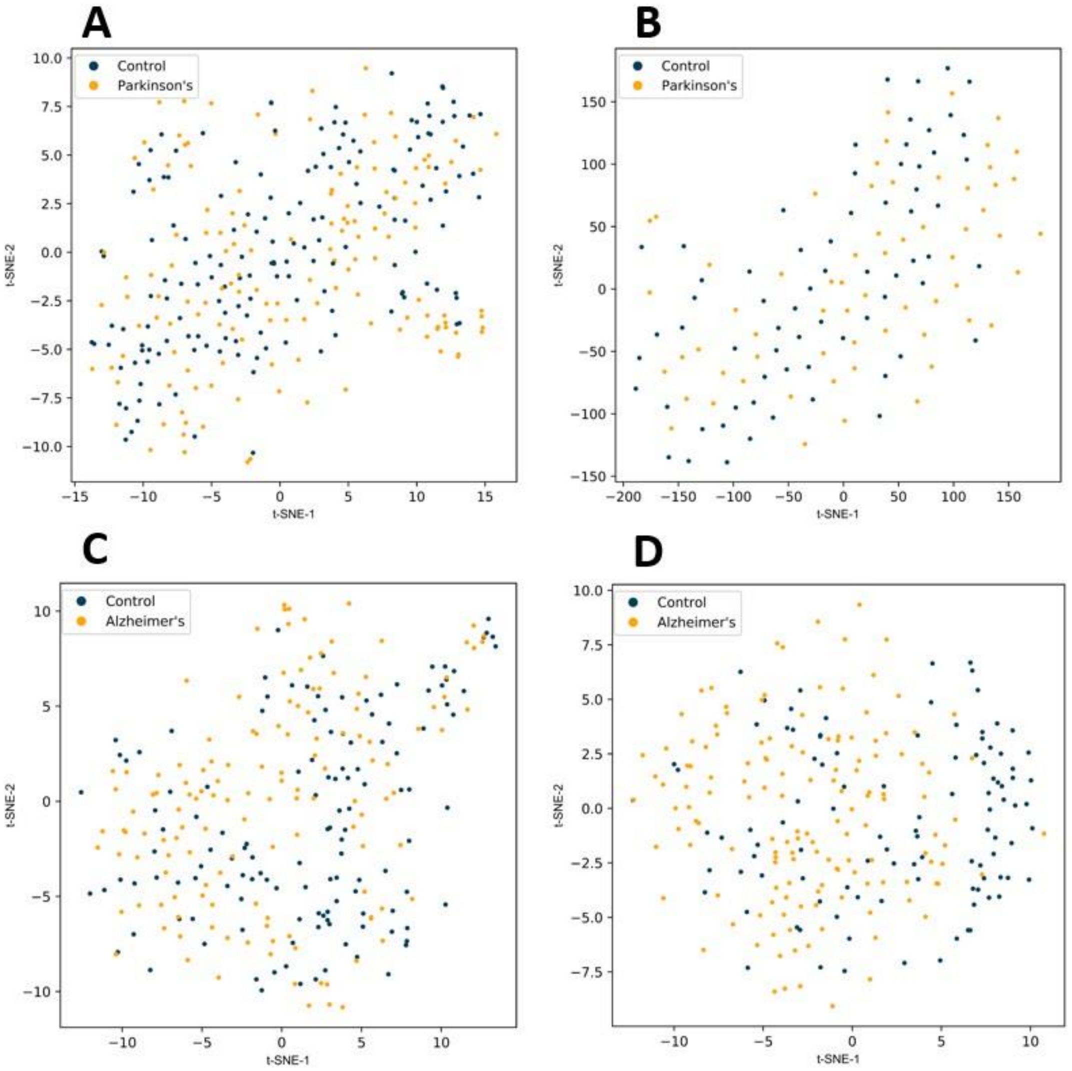

Supplementary Figure S1: t-SNE plots for training and test data of PD and AD. PD training (A) and test (B) datasets and AD training (C) and test (D) datasets show no outliers and no clear distinction between disease and control samples.

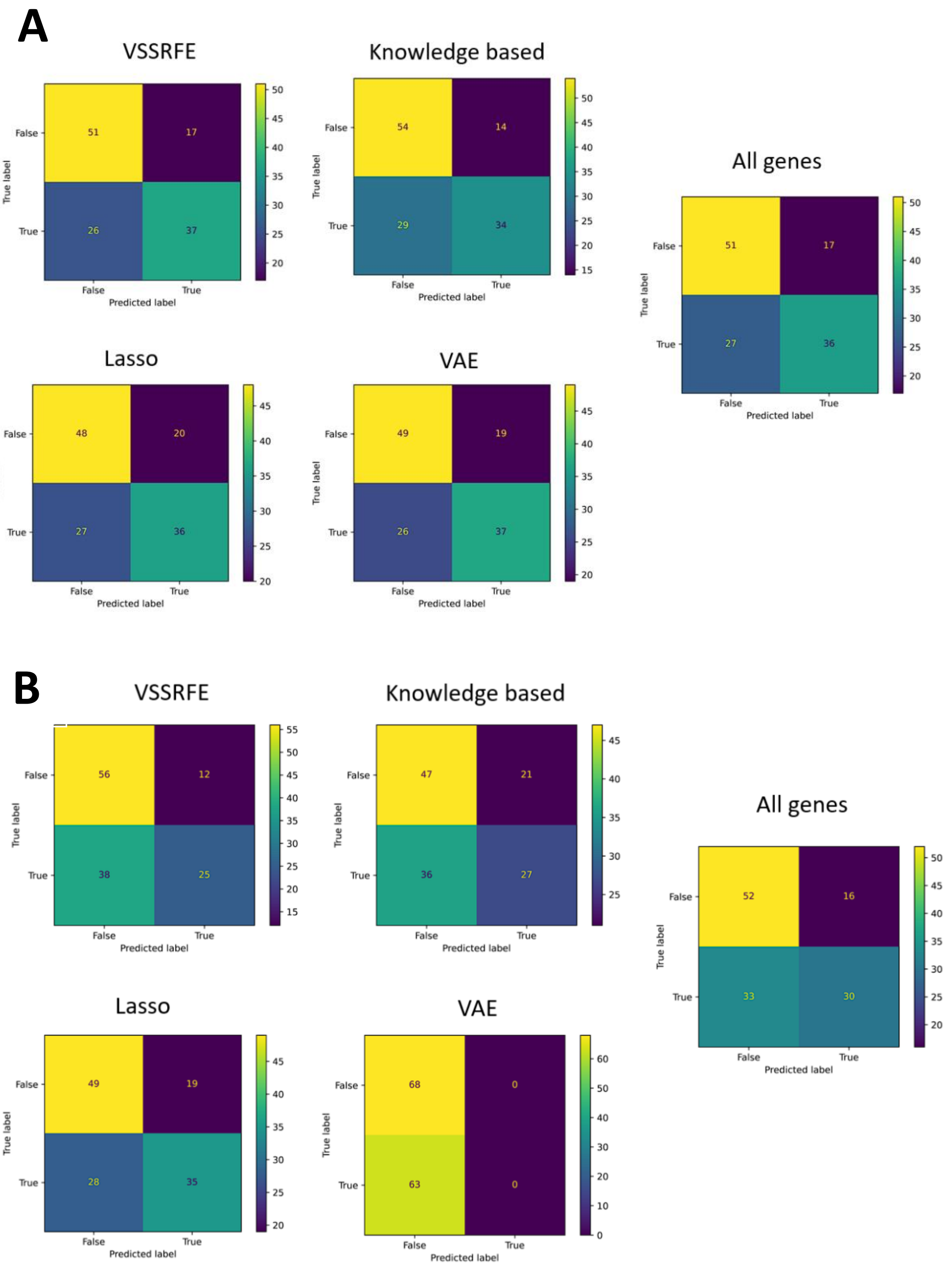
A
VSSRFE
51
17
26
37
Knowledge based
54
14
29
34
All genes
51
17
27
36
Lasso
48
20
27
36
VAE
49
19
26
37
B
VSSRFE
56
12
38
25
Knowledge based
47
21
36
27
All genes
52
16
33
30
Lasso
49
19
28
35
VAE
68
0
63
0
True label
Predicted label
False
True

C

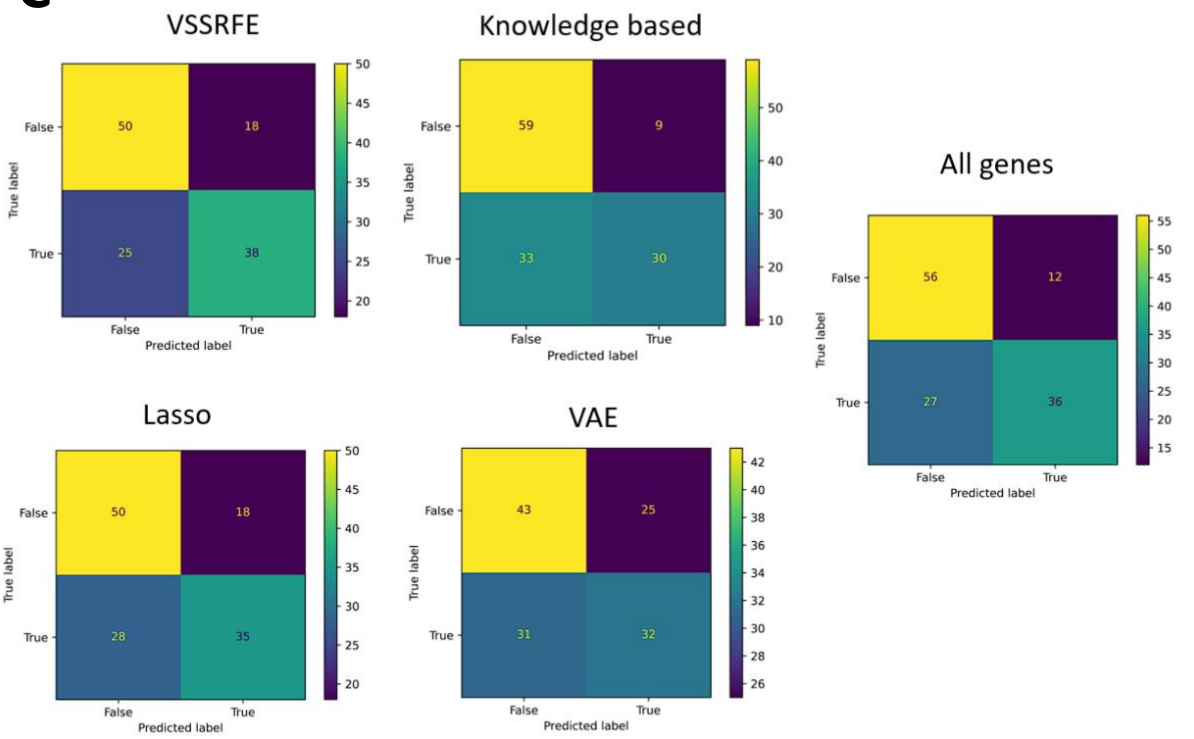
VSSRFE
True label
False 50 18
True 25 38
False True
Predicted label
Knowledge based
True label
False 59 9
True 33 30
False True
Predicted label
All genes
True label
False 56 12
True 27 36
False True
Predicted label
Lasso
True label
False 50 18
True 28 35
False True
Predicted label
VAE
True label
False 43 25
True 31 32
False True
Predicted label

D

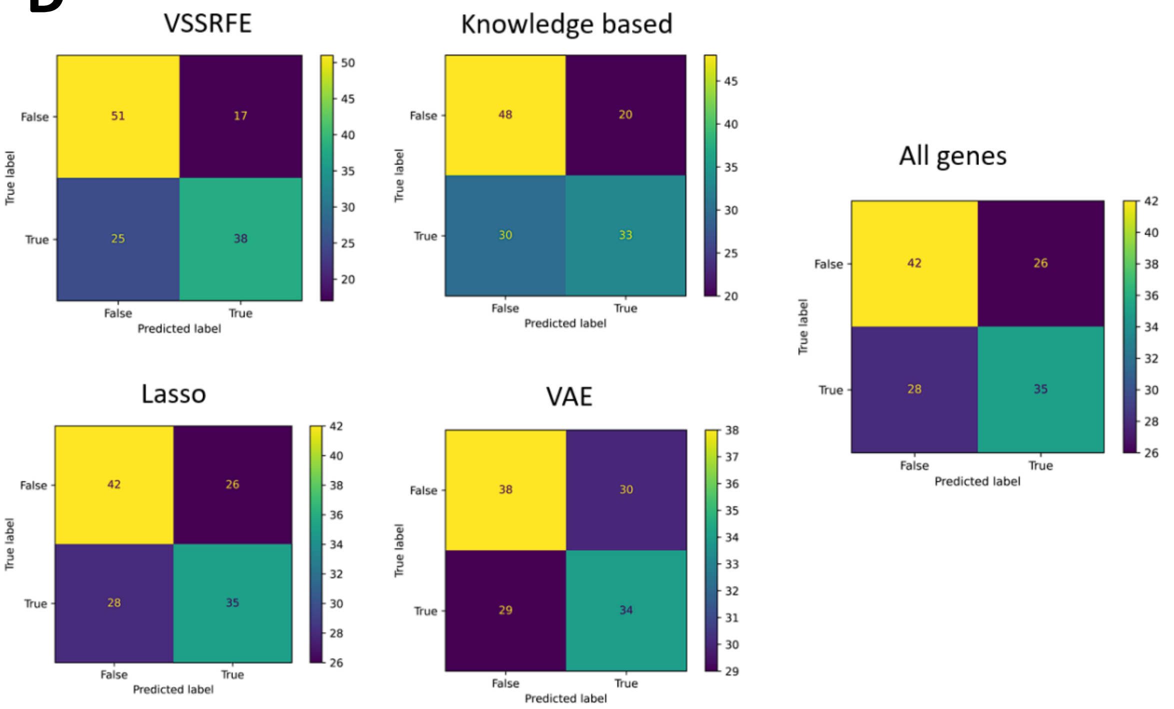
VSSRFE
True label
False 51 17
True 25 38
False True
Predicted label
Knowledge based
True label
False 48 20
True 30 33
False True
Predicted label
All genes
True label
False 42 26
True 28 35
False True
Predicted label
Lasso
True label
False 42 26
True 28 35
False True
Predicted label
VAE
True label
False 38 30
True 29 34
False True
Predicted label

E

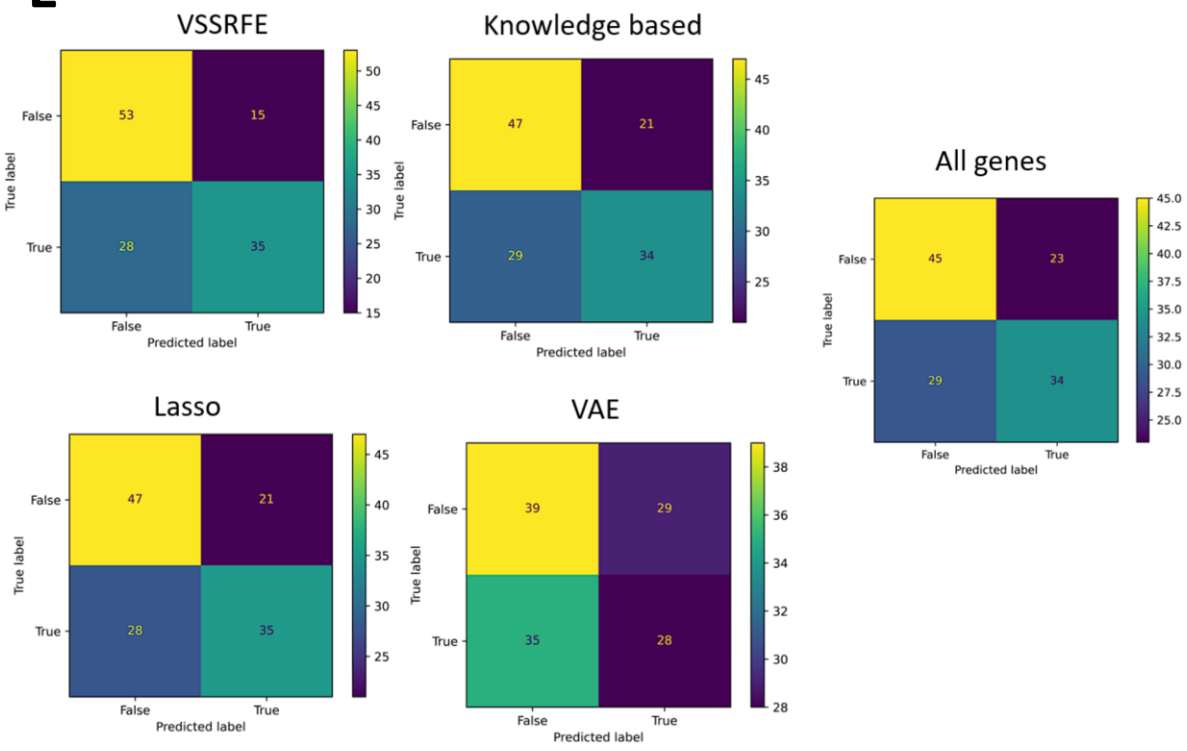
VSSRFE
Knowledge based
All genes
Lasso
VAE
True label
Predicted label
False
True
53
15
28
35
47
21
29
34
45
23
39
35
28

F

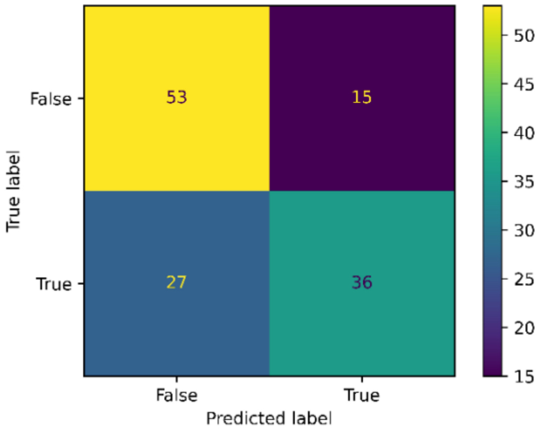
True label
Predicted label
False
True
53
15
27
36

G

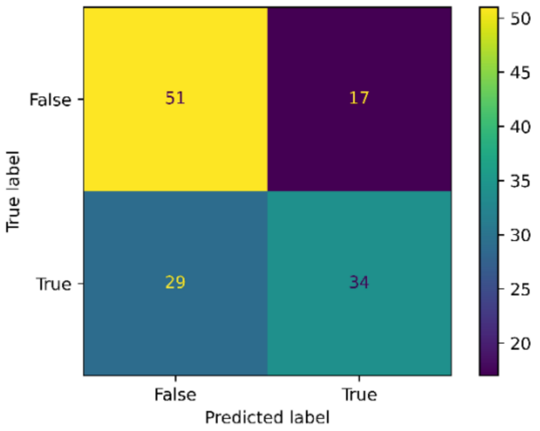
True label
Predicted label
False
True
51
17
29
34

Supplementary Figure S2. Confusion matrices summarising the performance of all classification models with each feature set on PD data. The models used are (A) linear regression, (B) SVM, (C) RF, (D) XGBoost, (E) MLP, (F) CNN, (G) VAE.

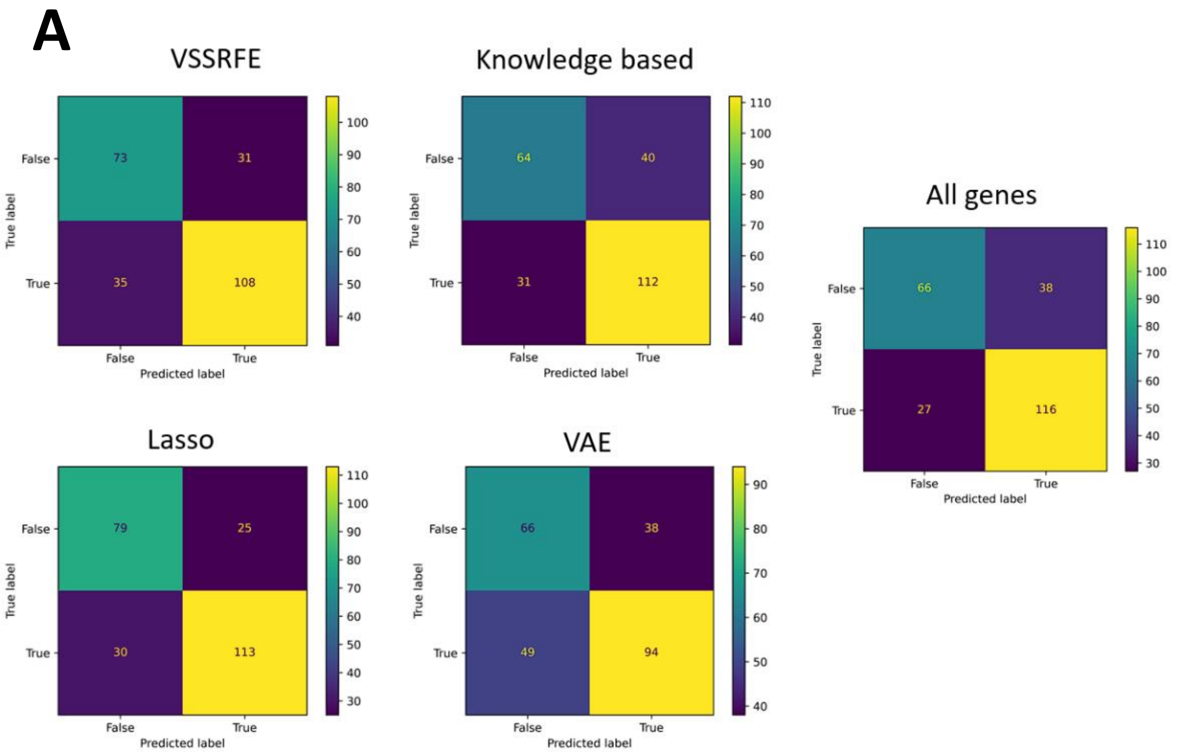

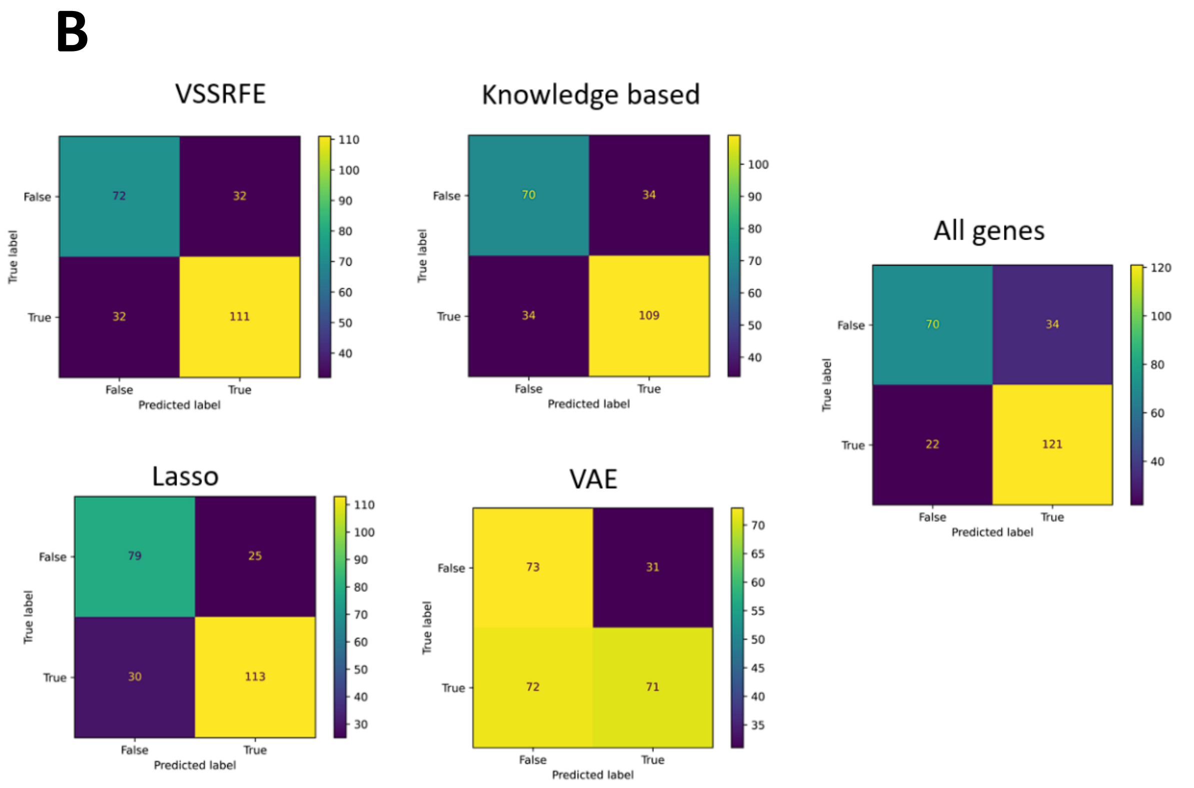

C

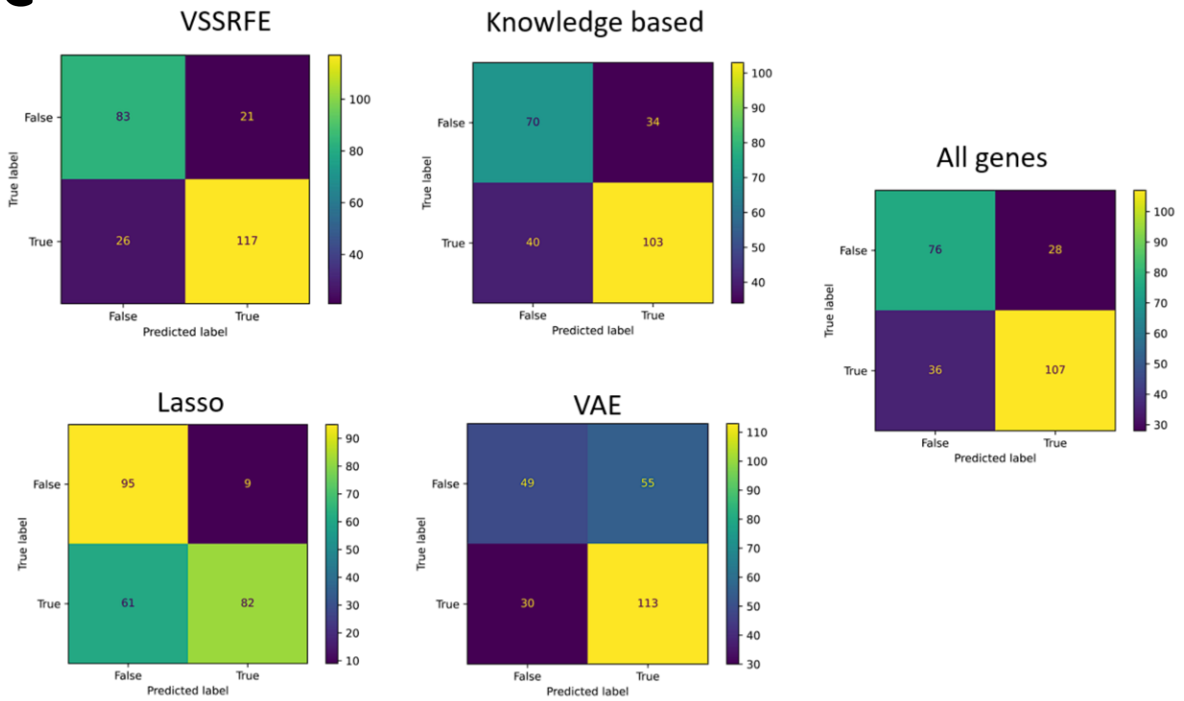
VSSRFE
83
21
26
117
Knowledge based
70
34
40
103
All genes
76
28
36
107
Lasso
95
9
61
82
VAE
49
55
30
113
True label
Predicted label
False
True

D

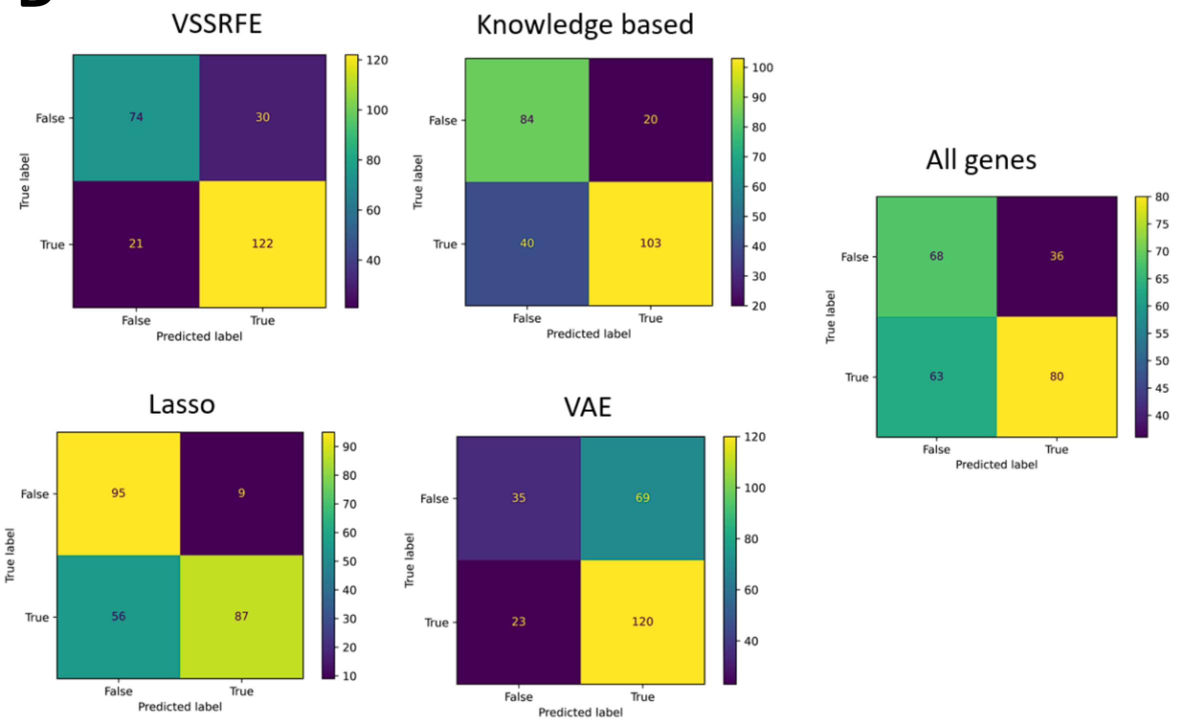
VSSRFE
74
30
21
122
Knowledge based
84
20
40
103
All genes
68
36
63
80
Lasso
95
9
56
87
VAE
35
69
23
120
True label
Predicted label
False
True

E

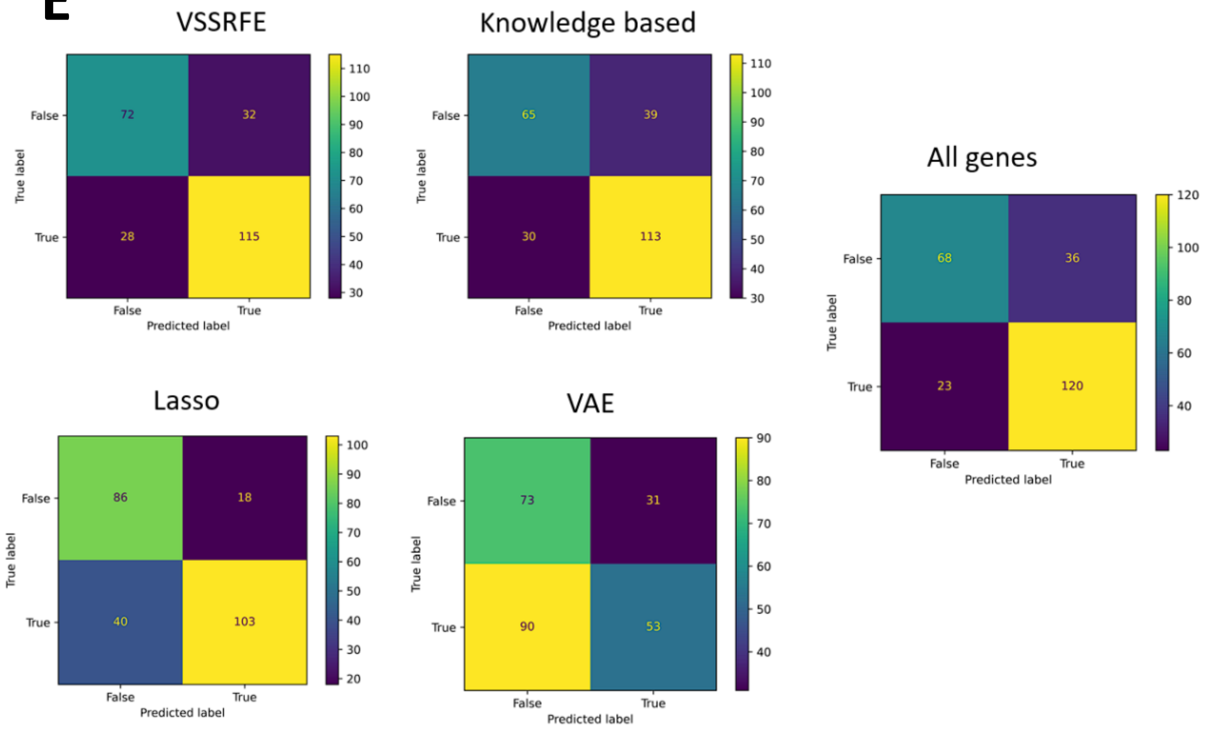
VSSRFE
72
32
28
115
Knowledge based
65
39
30
113
All genes
68
36
23
120
Lasso
86
18
40
103
VAE
73
31
90
53
True label
Predicted label
False
True

F

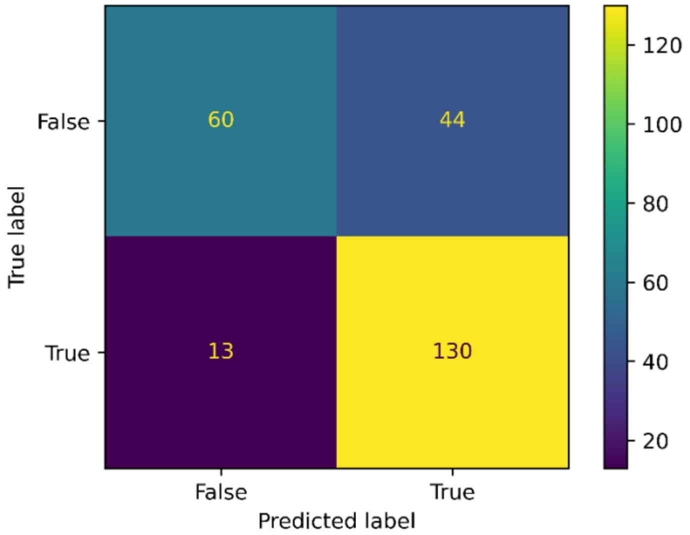
60
44
13
130
True label
Predicted label
False
True

G

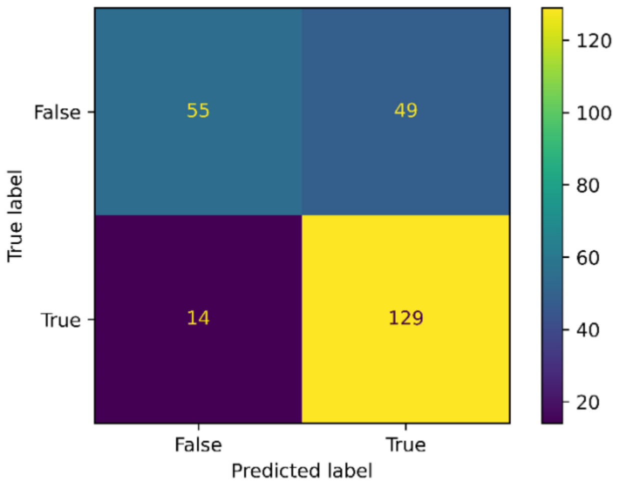

Supplementary Figure S3. Confusion matrices summarising the performance of all classification models with each feature set on AD data. The models used are (A) linear regression, (B) SVM, (C) RF, (D) XGBoost, (E) MLP, (F) CNN, (G) VAE.
